# Proglucagon-Derived Peptides Expression and Secretion in Rat Insulinoma INS-1 Cells

**DOI:** 10.3389/fcell.2020.590763

**Published:** 2020-11-10

**Authors:** Ana Acosta-Montalvo, Chiara Saponaro, Julie Kerr-Conte, Jochen H. M. Prehn, François Pattou, Caroline Bonner

**Affiliations:** ^1^INSERM, U1190, Lille, France; ^2^European Genomic Institute for Diabetes, Lille, France; ^3^University of Lille, Lille, France; ^4^Department of Physiology and Medical Physics, Royal College of Surgeons in Ireland, Dublin, Ireland; ^5^Chirurgie Endocrinienne et Métabolique, CHU Lille, Lille, France; ^6^Institut Pasteur de Lille, Lille, France

**Keywords:** INS-1 cells, bi-hormonal cells, insulin, glucagon, proglucagon, glicentin

## Abstract

Rat insulinoma INS-1 cells are widely used to study insulin secretory mechanisms. Studies have shown that a population of INS-1 cells are bi-hormonal, co-expressing insulin, and proglucagon proteins. They coined this population as immature cells since they co-secrete proglucagon-derived peptides from the same secretory vesicles similar to that of insulin. Since proglucagon encodes multiple peptides including glucagon, glucagon-like-peptide-1 (GLP-1), GLP-2, oxyntomodulin, and glicentin, their specific expression and secretion are technically challenging. In this study, we aimed to focus on glucagon expression which shares the same amino acid sequence with glicentin and proglucagon. Validation of the anti-glucagon antibody (Abcam) by Western blotting techniques revealed that the antibody detects proglucagon (≈ 20 kDa), glicentin (≈ 9 kDa), and glucagon (≈ 3 kDa) in INS-1 cells and primary islets, all of which were absent in the kidney cell line (HEK293). Using the validated anti-glucagon antibody, we showed by immunofluorescence imaging that a population of INS-1 cells co-express insulin and proglucagon-derived proteins. Furthermore, we found that chronic treatment of INS-1 cells with high-glucose decreases insulin and glucagon content, and also reduces the percentage of bi-hormonal cells. In line with insulin secretion, we found glucagon and glicentin secretion to be induced in a glucose-dependent manner. We conclude that INS-1 cells are a useful model to study glucose-stimulated insulin secretion, but not that of glucagon or glicentin. Our study suggests Western blotting technique as an important tool for researchers to study proglucagon-derived peptides expression and regulation in primary islets in response to various metabolic stimuli.

## Introduction

The loss of functional insulin-producing beta-cells is a hallmark of diabetes, therefore understanding the cellular biology of the pancreas is crucial. In this context, primary islets represent a critical resource for investigators studying diabetes. However, the production of islets for research is contingent on the availability of rodent and/or donor pancreata, and the process of islet isolation is also technically demanding and expensive (Kaddis et al., 2009; Saponaro et al., 2020). This creates a unique set of challenges, with *in vitro* studies on diabetes often reliant on various rodent and human clonal cell lines such as INS-1, MIN6, RIN, HIT, βTC, EndoC-βH1, and alpha TC (Chick et al., 1977; Gazdar et al., 1980; Santerre et al., 1981; Miyazaki et al., 1990; Asfari et al., 1992; Ohgawara et al., 1995; Poitout et al., 1995; Bonner et al., 2010, 2015; Skelin et al., 2010; Weir and Bonner-Weir, 2011; Tsonkova et al., 2018). One commonly used tumor cell line is the INS-1 cells, derived from a rat insulinoma induced by X-ray irradiation, which displays many characteristics of pancreatic beta-cells including high insulin content and their ability to respond to glucose fluxes (Asfari et al., 1992; Bonner et al., 2010; Jhun et al., 2013; Eto et al., 2014; Heaslip et al., 2014). Although islet cells expressing more than one hormone have been identified during pancreatic development (De Krijger et al., 1992), it also appears that INS-1 cells co-express insulin and glucagon, thus revealing the bi-hormonal features of this cell line (Bollheimer et al., 2005; Wang et al., 2015). The transcription factor Nkx6.1 is required for the differentiation and maturation of beta-cells and has been shown to suppress glucagon expression in bi-hormonal INS-1 cells (Schisler et al., 2005). Indeed, bi-hormonal INS-1 cells have reduced expression of Nkx6.1 and do not express alpha cell markers, thus suggesting the immature state of these cells (Watada et al., 2000; Schisler et al., 2005; Rezania et al., 2013; Taylor B. L. et al., 2013; Wang et al., 2015). The bioactive hormone products of proglucagon and proinsulin protein are controlled by the post-translational modification of two prohormone convertases (PC) PC1/3 and PC2 (Bailyes et al., 1991; Itoh et al., 1996). The cleavage of proglucagon by PC1/3 results in the production of GLP-1, GLP-2, and glicentin as well as oxyntomodulin (Furuta et al., 1997, 2001; Pocai, 2012). PC2 cleaves proglucagon protein to produce major proglucagon fragment, glicentin related pancreatic polypeptide (GRPP), and glucagon peptides. Like beta-cells, INS-1 cells also express PC1/3 and PC2 enzymes (Marzban et al., 2004; Wang et al., 2015), thus, suggesting that the proglucagon protein expressed in bi-hormonal INS-1 cells may be cleaved to produce different proglucagon derived peptides. It is known that chronic glucose-stimulation reduces *insulin* gene and protein expression in INS-1 cells (Marie et al., 1993; Olson et al., 1998), however, whether it affects proglucagon-derived peptides expression and regulation is unknown. Therefore, the aim of this study was to investigate proglucagon-derived peptides (PDPs) expression and regulation by glucose in INS-1 cells using several complementary antibody-based techniques.

## Materials and Methods

### Cell Culture

Rat insulinoma INS-1 cells (generated by Prof. Claes Wolheim, University of Geneva, Switzerland) were cultured RPMI 1640 medium containing 11 mM glucose (Gibco, Cat No. 11879020, Grand Island, NY, United States) supplemented with 10% fetal bovine serum (FBS; Eurobio, Cat No. CVFSVF06-01, Les Ulis, France), 100 U/ml penicillin-streptomycin (P/S; Gibco, Cat No. 15140-122, Grand Island, NY, United States), 10 mM HEPES (Sigma-Aldrich, Cat No. H4034-500G, St. Louis, MO, United States), 1 mM sodium pyruvate (Gibco, Cat No. 11360-039, Grand Island, NY, United States) and 50 μM β-mercaptoethanol (Gibco, Cat No. 21985-023, Grand Island, NY, United States). Human embryonic kidney (HEK293) cells (CRL-1573, ATCC) were cultured in DMEM GlutaMAX 5.5 mM glucose supplemented with 10% FBS (Eurobio, Cat No. CVFSVF06-01, Les Ulis, France) and 100 U/mL penicillin/streptomycin (Gibco, Cat No. 15140-122, Grand Island, NY, United States) according to the manufacturer’s recommendations.

### Immunofluorescence Imaging

INS-1 cells (5 × 10^4^ cells/well) were cultured for 24 h in a 24-well plate, each containing a sterile glass coverslip with 1 mL of RPMI 1640 supplemented with 11 mM glucose, 10% FBS, 100 U/ml P/S, 10 mM HEPES, 1 mM sodium pyruvate and 50 μM β-mercaptoethanol. Cells were fixed in 4% PFA-PBS at room temperature for 1 h and permeabilized with phosphate buffer saline (PBS; Gibco, Cat No. 14190136, Paisley, United Kingdom) supplemented with 1% bovine serum albumin (BSA; Roche, Cat No. 10735094001, Mannheim, Germany) and 0.1% Triton 100× (Sigma-Aldrich, Cat No. X100-100 mL, St. Louis, MO, United States) for 15 min. Human pancreatic slices were deparaffinized following a standard protocol. Heat-mediated antigen retrieval was performed with citrate buffer pH = 6 in the microwave (650 W) for 9 min. Cells and tissues were incubated with serum-free protein block (Dako, Cat No. X0909, Santa Clara, CA, United States) for 30 min at room temperature. Single or double immunofluorescence staining was performed at room temperature for 2 h with the following antibodies: guinea pig polyclonal anti-insulin (Dako, Cat No. A0564, Carpinteria, CA, United States) 1:500 dilution in PBS; rabbit monoclonal anti-glucagon (Abcam, Cat No. 92517, CA, United States) 1:3000 dilution in PBS for cells and 1:1000 dilution in PBS for pancreatic islets and, mouse monoclonal anti-HPA2 (Novus Biologicals, Cat No NBP1-18950) 1:100 dilution in PBS. Secondary Alexa Fluor antibodies (anti-mouse AF-594, Invitrogen, Cat. No. A11032, Eugene, OR, United States; anti-rabbit AF-594, Invitrogen, Cat. No. A11012, Eugene, OR, United States; anti-rabbit AF-488, Invitrogen, Cat. No. A21206, Eugene, OR, United States; anti-guinea pig AF-488, Abcam, Cat. No. ab150185, CA, United States) were diluted 1:500 in PBS and applied for 1 h at room temperature. Finally, nuclei were counterstained with DAPI (Vectashield, Vector Laboratories, Cat No. H-1200-10, Burlingame, CA, United States) and mounted with Dako Fluorescence Mounting Medium (Dako, cat No. 53023, Carpinteria, CA, United States). Images were acquired using the confocal microscope Zeiss LSM 710 with Airyscan super-resolution module (Zeiss, Germany). The images were obtained using a 40× objective (Jena, Germany) with immersion oil. The images were processed and adjusted using ImageJ, version 2.0.0-RC-43/1.50e.^1^

### Islet Isolation and Culture

Human islets were isolated as previously described (Kerr-Conte et al., 2010), and cultured in CMRL media 1066 (Gibco, Cat No. 041-95397M, Paisley, United Kingdom) supplemented with 0.625% human albumin (Vialebex, Les Ulis, France) and 100 U/ml Pen/Strep. Pancreases from two Wistar rats were used for islet isolation. The rats were anesthetized and after the procedure, were sacrificed by cervical dislocation. Each rat was laid with the abdominal side facing up and the skin was sterilized with 70% ethanol. The pancreas was exposed and infused with cold enzyme collagenase P (Roche, Cat No. 11213865001, Mannheim, Germany) via the common bile duct. The pancreas was removed and collected in a 50 ml tube containing 2 ml of enzyme collagenase, and digested in a water bath at 37°C for 8–10 min. Enzymatic digestion was stopped by adding cold Hanks’ Balanced Salt solution (Gibco, Cat No. 14065-049, Paisley, United Kingdom) containing 1% of albumin. Islet purification was performed by density gradient with polysucrose 1,132/1,108/1,096/1,069/1,000 (Mediatech, Cat No. 99-662-CVS, Miami, FL, United States). Islets showed > 90% purity (endocrine versus exocrine tissue), and cultured in RPMI-1640 glucose-free medium supplemented with 5,5 mM glucose, 10% FBS, and 1% P/S for 18 h before stimulation.

### Western Blot Analysis

Human islets and Wistar rat islets (2000 islet equivalents (IEQ), were harvested in 80 μL of lysis buffer containing 20 mM Tris-Acetate, 0.27 M sucrose, 1% Triton X-100, 1 mM EDTA, 1 mM EGTA, 50 mM Sodium Fluoride and 10 mM Beta glycerophosphate; INS-1 cells (1 × 10^6^ cells) were harvested with 80 μL of CelLytic M reagent (Sigma-Aldrich, Cat No. C2978, St. Louis, MO, United States). Lysis buffer was supplemented with proteinase inhibitors (Sigma-Aldrich, Cat No. P8340, St. Louis, MO, United States) and phosphatases inhibitors PhosSTOP (Roche, Cat No. 04906837001, Mannheim, Germany) according to the manufacturer’s instructions. After sonication, insoluble material was removed by centrifugation at 12000 rpm for 20 min at 4°C. Protein concentration was determined using the Pierce BCA protein assay kit (Thermo-Fisher Scientific, Cat No. 23225, Rockford, IL, United States). Total protein from INS-1 cell lysates (15 μg), Wistar rat islet lysates (25 μg) and human islet lysates (5 μg) were supplemented with 4× Laemmli SDS sample buffer (Alfa Aesar, Cat No. J60015, Kandel, Germany), denatured at 95°C for 5 min and separated in a premade gel (Thermo-Fisher Scientific, Cat No. NW04120BOX, Carlsbad CA, United States) or homemade 20% SDS-PAGE gel (Supplementary Table 1); proteins were transferred to nitrocellulose membranes using the Dry iBlot (Thermo-Scientific, Carlsbad, CA, United States) transfer system for 6 min at 25V. The membranes were blocked with 5% BSA in Tris Buffer Saline (TBS) containing 0.1% of Tween 20 (Sigma-Aldrich, Cat No. P1379-500 ML, St. Louis, MO, United States; TBST) for 60 min at room temperature and washed 3 times with TBST for 15 min. Membranes were incubated overnight at 4°C with rabbit monoclonal anti-insulin 1:2500 dilution (Abcam, Cat No. ab181547, CA, United States) or rabbit monoclonal anti-glucagon 1:2500 dilution (Abcam, Cat No. ab92517, CA, United States), diluted in 5% BSA-TBST. After washing, membranes were incubated with the anti-rabbit HRP-conjugated or anti-mouse HRP-conjugated antibody (GE Healthcare Life Science Cat No. NA934-1 ML, Little Chalfont, United Kingdom), diluted 1:10000 in 5% BSA-TBST for 1 h at room temperature. The membranes were washed and developed with ECL Plus according to the manufacturer’s instructions (GE Healthcare Life Science, Cat No. RPN2236, Italy). Digital images were taken and analyzed with the Amersham Imager 600. Mouse monoclonal anti-beta actin (Sigma-Aldrich, Cat No. A5441-2 ML, St. Louis, MO, United States) and rabbit polyclonal anti-gapdh antibodies (Sigma-Aldrich, Cat No. G8795, St Louis, MO, United States) were used as loading controls.

### Real-Time PCR

Total RNA was extracted from INS-1 cells using the RNeasy Mini Kit (QIAGEN, cat No. 74104, Hilden, Germany). First-strand cDNA synthesis was performed using 0,5 μg total RNA as a template and iScript Select cDNA synthesis kit (Biorad, Cat No. 1708897BUN, Hercules, CA, United States) according to manufacturer’s instructions. Real-time PCR was performed using the Bio-Rad MyiQ Single-Color Real-Time PCR Detection System and the Bio-Rad IQ SYBR Green Supermix (Bio-Rad Laboratories, cat No. 172-5274, United States) as previously described (Bonner et al., 2015). Primers for *proinsulin, proglucagon*, *Pcsk1/3*, *Pcsk2, and Rlp27* genes were designed with Primer3 software (Supplementary Table 2); the final concentration used for all the primers was 0,5 pmol/uL (0,25 pmol/uL of primer forward and 0,25 pmol/uL of primer reverse). The cDNA was diluted 1:10 and 2 μL was used in 10 μL final volume for each reaction. The thermal cycler protocol was: 95°C for 3 min, followed by 40 cycles, 1 cycle at 95°C for 10 s (denaturation) and 60°C for 30 s (annealing). A melt curve analysis was performed as qPCR reaction control. Gene expression was normalized to *Rlp27* housekeeping gene.

### Glucose Stimulated Hormone Secretion

INS-1 cells (2 × 10^5^ cells/well) were seeded in a 12 well plate with 11 mM glucose RPMI 1640 media. After 48 h, cells were washed twice with KRB buffer (135 mM NaCl, 5 mM KCl, 1 mM MgSO4, 1,2 mM K2HPO4, 10 mM HEPES, 2,6 mM CaCL2, 5 mM NaHCO3; pH: 7.4) supplemented 0,5% BSA. For glucose-stimulated hormone secretion, cells were washed and preincubated for 1 h with 0,5 mM glucose in KRB buffer and stimulated for 1 h with 2,5 mM and 20 mM glucose to determine insulin secretion, and with 1 mM, 6 mM 11 mM and 20 mM glucose to determine glicentin and glucagon secretion. Supernatants were collected in and cellular content was extracted from INS-1 cells with 0,5 mL of ethanol acid. Supernatant and cellular content were stored at −20°C before hormone measurement.

### Hormone Measurements

Insulin secretion (non-diluted) and cell content (1:5 dilution with calibrator 0) were measured using Mercodia Rat Insulin Elisa Kit (Mercodia AB, Cat No. 10-1250-01, Uppsala, Sweden). The detection limit is < 0,15 μg/L as determined with the methodology described in their manual. Glucagon secretion (non-diluted) and cell content (1:100 dilution with calibrator 0) were measured using Mercodia Glucagon Elisa 10 μL Kit (Mercodia AB, Cat No. 10-1281-01, Uppsala, Sweden). The detection limit is 1.5 pmol/L as determined with the methodology described in their manual. Glicentin secretion (1:5 dilution with calibrator 0) and cell content (1:100 dilution with calibrator 0) were measured using the Mercodia Rat Glicentin Elisa Kit (Mercodia AB, Cat No. 10-1275-01, Uppsala, Sweden). The detection limit is 5 pmol/L as determined with the methodology described in their manual. All ELISA procedures were performed according to the manufacturer’s instructions. The Mercodia Glucagon ELISA (10-1281-01) is validated with a simultaneous assay protocol, however in our situation with high levels of glicentin Mercodia proposed an alternative sequential protocol, originally developed for the IVD labeled Mercodia Glucagon ELISA (10-1271-01). This sequential protocol abolishes even low cross-reactivity with glicentin that might interfere when glicentin levels are exceptionally high (Roberts et al., 2018), by adding an extra washing step that removes unspecific bout material from the antibody-coated plate.

### Flow Cytometry Analysis

INS-1 cells (1 × 10^6^ cells/condition) were permeabilized and fixed with BD Cytofix/cytoperm buffer (BD biosciences, Cat No. 554714, San Diego, CA, United States) according to manufacturer’s instructions. Cells were incubated overnight at 4°C with the following primary antibodies diluted in cytoperm buffer: rabbit monoclonal anti-insulin 1:400 (Abcam, Cat No. ab181547, CA, United States) and mouse monoclonal anti-glucagon (Sigma-Aldrich, Cat No. G2654, Israel) 1:400 dilution in PBS. After washing, cells were incubated for 1 h at room temperature and protected from the light with the following secondary antibodies diluted 1:500 in cytoperm buffer: Alexa Fluor anti-rabbit AF-488 (Invitrogen, Cat. No. 21206, Eugene, OR, United States) and anti-mouse, AF-594 (Invitrogen, Cat. No. 11032, Eugene, OR, United States). Isotype controls were used to set-up negative gate: Rabbit IgG AF-488 1:400 dilution (Thermo Fischer Scientific, N°A21206, Carlsbad, CA, United States; and BD Horizon PE-CF594 Mouse IgG, k isotype control 1:400 dilution (BD bioscience, Cat No. 562292, San Diego, CA, United States). Cells were re-suspended in 500 μL FACS buffer (PBS+10%FBS). Flow cytometry data were acquired on LSR Fortessa X20 (BD Biosciences) and analyzed using Kaluza software.

### Statistical Analysis

Data are expressed as means ± SEM, one-way ANOVA, and Tukey’s *post hoc* tests were used for multiple comparisons. Statistical analyses were performed using GraphPad PRISM 7.0 (GraphPad Software, La Jolla, CA, United States). Differences were considered significant at *p* < 0.05.

## Results

### Validation of the Anti-glucagon Antibody (Abcam) Reveals That Is Not Specific Solely for Glucagon but Also Detects Proglucagon and Glicentin

**FIGURE 1:**
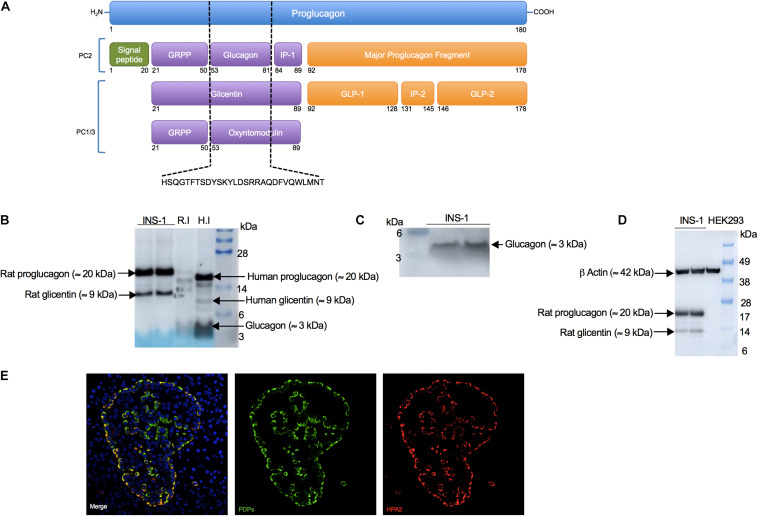
Anti-glucagon antibody (Abcam) detects proglucagon-derived peptides (proglucagon, glucagon and glicentin) in INS-1 cells, primary islets (positive controls) but not in HEK293 cells (negative control). **(A)** Scheme of the proglucagon derived peptides, which share the entire glucagon sequence (dashed black lines). Scheme adapted from Rouille et al. (1994) and Wewer Albrechtsen et al. (2016). **(B)** Western blot analysis of proglucagon (≈ 20 kDa) and glicentin (≈ 9 kDa) in INS-1 cells in addition to glucagon (≈ 3 kDa) observed in rat islets (R.I) and human islets (H.I) (positive controls), using an anti-glucagon antibody (Abcam, Cat No. ab92517). Fifteen μg of INS-1 cells, 25 μg of rat islets or 5 μg of human islets protein lysates were loaded. Exposure time for proglucagon proteins: 40 s **(C)** Western blot analysis of glucagon (≈ 3 kDa) in INS-1 cells after a longer exposure. Exposure time for glucagon: 60 s. **(D)** Western blot analysis of proglucagon (≈ 20 kDa) and glicentin (≈ 9 kDa) in INS-1 and in HEK293 cells (negative control). Exposure time: 30 s. **(E)** Representative images of immunofluorescence staining for proglucagon-derived peptides (PDPs; green) using anti-glucagon antibody (Abcam, Cat No. ab92517) and HPA2 (red) using an anti-HPA2 antibody (mouse monoclonal, clone DHIC2-2B4, Cat. No. MABS2045) performed on paraffin-embedded human pancreatic sections.

Earlier studies suggested that INS-1 cells co-express insulin and glucagon proteins (Bollheimer et al., 2005; Wang et al., 2015). Since proglucagon, glicentin and oxyntomodulin are peptides that share the entire amino acid sequence of glucagon (Figure 1A), we envisaged that it would be difficult to find an antibody specific solely for glucagon. To determine if this was the case, we performed Western blot analysis in INS-1 cells and rat and human islet lysates (positive controls for glucagon). We found that the rabbit monoclonal anti-glucagon antibody from Abcam (Cat No. ab92517) detected proglucagon (≈ 20 kDa), glicentin (≈ 9 kDa) and glucagon (≈ 3 kDa) proteins, according to their molecular weight, in agreement with previous studies (Rouille et al., 1994; Dey et al., 2005; Piro et al., 2010). Under these exposure conditions (Exposure time: 40 s), we did not detect a signal for glucagon (≈ 3 kDa) in INS-1 cells, but we observed an intense signal for proglucagon and glicentin peptides (Figure 1B). Based on this, we cut the membrane to determine if a longer exposure time would enable us to detect glucagon (≈ 3 kDa). Indeed, after 60 s exposure, we observed that INS-1 cells expressed glucagon (≈ 3 kDa) (Figure 1C), which was absent in the human kidney embryonic cell line, HEK293, as expected, thus serving as a negative control (Figure 1D). Using the same anti-glucagon antibody, we performed immunofluorescence imaging in human pancreatic sections, which showed co-localization of proglucagon-derived peptides (PDPs) and HPA2, a specific marker of alpha cells (Dorrell et al., 2008; Figure 1E). However, it is important to remember that immunofluorescence-based techniques cannot discriminate between proteins that share the same amino acid sequence, therefore we will refer to proglucagon-derided peptides (PDPs) (proglucagon, glicentin, and glucagon) detected by immunofluorescence imaging and cytometry analysis.

### Bi-Hormonal INS-1 Cells Co-express Insulin and PDPs Proteins

**FIGURE 2:**
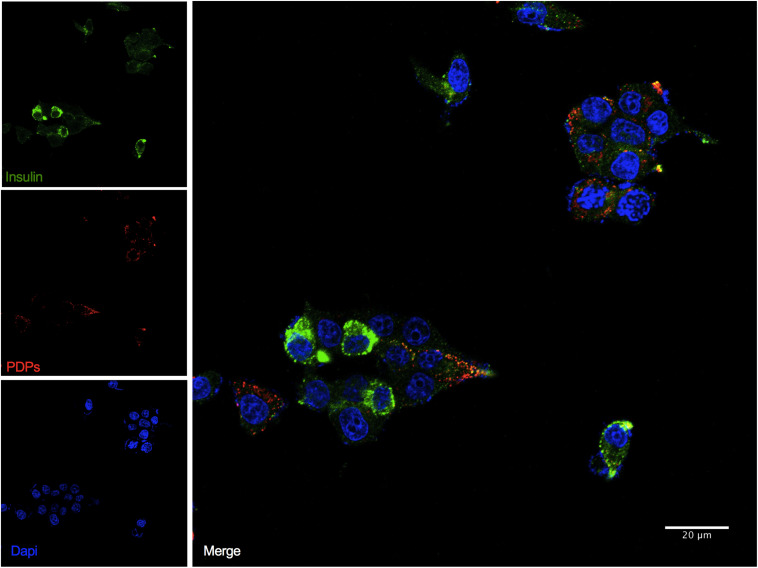
INS-1 cells express insulin and glucagon proteins. Representative images of immunofluorescence confocal imaging (Zeiss LSM 710) for insulin (green) using an anti-insulin antibody (Dako, Cat No. A0564) and PDPs (red) using an anti-glucagon antibody (Abcam, Cat No. 92517) performed on formalin-fixed INS-1 cells attached to coverslips. Scale bar: 20 μm. The experiment was performed in triplicate and repeated two times.

To determine if a subpopulation of INS-1 cells were co-expressing insulin and PDPs, we performed dual-indirect immunofluorescence analysis with our validated anti-glucagon antibody. We demonstrated that all INS-1 cells were insulin-positive as expected (Wang et al., 2015). Although insulin was heterogeneous, we observed that a subset of INS-1 cells also co-expressed PDPs (proglucagon, glicentin, and glucagon) (Figure 2).

### INS-1 Cells Secrete Insulin in Response to Acute Glucose Stimulation and Regulates *Ins* Gene and Protein Expression in Response Chronic Glucose Stimulation

**FIGURE 3:**
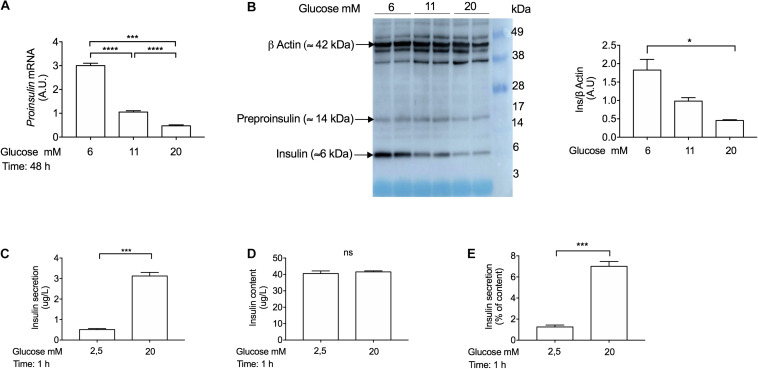
Glucose coordinately regulates proinsulin (*Ins*) gene expression, insulin protein expression, and insulin secretion in INS-1 cells. **(A)** Real-time PCR analysis of *proinsulin* gene expression in INS-1 cells cultured at 6 mM, 11 mM, and 20 mM glucose for 48 h. Cells incubated with 11 mM glucose were used as control and were set to an arbitrary value of 1. Gene expression was normalized to *Rlp27* gene. **(B)** Western blot analysis of insulin using anti-insulin antibody (Abcam, Cat No. ab181547; ≈ 6 kDa) expression in INS-1 cells incubated at 6 mM, 11 mM, and 20 mM glucose for 48 h. Fifteen μg of protein lysates were loaded in a pre-made gel (14–20%). β-actin was used as a loading control. Exposure time for insulin: 90 s. Exposure time for β-actin: 3 s. **(C)** Insulin secretion, **(D)** insulin content and **(E)** insulin secretion expressed as percentage of content from INS-1 cells. Insulin secretion and content were measured by ELISA kit (Mercodia, Cat No.10-1250-01). Cells were stimulated with different glucose concentrations in Krebs-Ringer modified buffer (KRB) for 1 h. Data are expressed as means ± SEM Specific *p*-values: *p < 0.05, ***p < 0.001. One-way ANOVA and Tukey’s *post hoc* tests were used. The experiment was performed in duplicate wells and it was repeated twice.

To determine that INS-1 cells were functional in response to glucose stimulation in our laboratory, we performed gene expression analysis, Western blotting and glucose-stimulated-insulin-secretion (GSIS) experiments. To confirm that INS-1 actually synthesizes *Ins* gene and protein, we incubated the cells with different glucose concentrations (6 mM, 11 mM, and 20 mM) for 48 h. Of note, INS-1 cells were cultured in 11 mM glucose, which is the required glucose concentration to maintain these cells in culture (Ullrich et al., 1996). In agreement with a previous report (Olson et al., 1998), we showed that chronic treatment with high-glucose concentrations (20 mM) decreased *Ins* gene (Figure 3A) and protein expression (Figure 3B), compared to low-glucose concentrations (6 mM). Using Western blot analysis, we observed that INS-1 cells express pre-proinsulin (≈ 14 kDa) and insulin (≈ 6 kDa) proteins (Figure 3B). To determine whether INS-1 cells were responsive to a glucose challenge, insulin secretion was measured using an ultrasensitive rat insulin ELISA kit (Mercodia, Cat No 10-1250-01). In order to enhance the sensitivity of INS-1 cells to a high-glucose challenge, the cells were pre-incubated in low-glucose medium (0,5 mM) for 1 h, and further stimulated with 2,5 mM or 20 mM glucose in KRB buffer for 1 h, as previously described (Asfari et al., 1992; Ullrich et al., 1996; Wang et al., 1998). Indeed, INS-1 cells secreted insulin in response to high-glucose concentrations (20 mM) (Figures 3C–E), thus validating INS-1 cells as a useful model to study the mechanisms of insulin secretion reported here and elsewhere (Asfari et al., 1992; Ullrich et al., 1996; Olson et al., 1998; Wang et al., 2000; Merglen et al., 2004; Bonner et al., 2010, 2011).

### Chronic Treatment With High-Glucose Concentrations Decreases the Number of Bi-Hormonal INS-1 Cells

**FIGURE 4:**
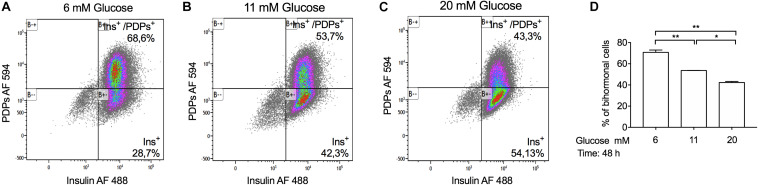
Chronic glucose treatment with a high-glucose concentration (20 mM) decreases the percentage of bi-hormonal INS-1 cells. Representative flow cytometry dot plot graphs, showing the percentage of insulin and PDP (proglucagon-derived peptides: proglucagon, glicentin, and glucagon) positive INS-1 cells incubated at **(A)** 6 mM, **(B)** 11 mM, and **(C)** 20 mM glucose for 48 h. **(D)** Quantification of the percentage of a population of bi-hormonal INS-1 cells. Data are expressed as means ± SEM Specific *p*-values: *p < 0.05, **p < 0.01. One-way ANOVA and Tukey’s *post hoc* tests were used. The experiment was performed in duplicate and it was repeated twice.

Several studies have shown that glucose plays an important role in beta-cell differentiation in the mouse and human pancreas and that beta-cells express other pancreatic hormones such as glucagon, under hyperglycemic conditions (Schuit et al., 2002; Talchai et al., 2012; Brereton et al., 2014). To study the chronic effect of hyperglycemia on the number of insulin and proglucagon positive-cells (bi-hormonal cells), we stimulated INS-1 cells with low-glucose (6 mM), basal glucose (11 mM) and high-glucose concentrations (20 mM) for 48 h. Subsequently, we performed flow cytometry analysis to determine the percentage of insulin, proglucagon, and double-positive-cells. We observed that 97% of INS-1 cells were insulin-positive in all glucose conditions tested, including single and double-positive-cells (Figures 4A–C). We did not observe INS-1 cells positive for proglucagon protein alone, in any of the conditions tested. The number of double-positive-cells increased in INS-1 cells incubated with low-glucose (68,59%) compared to basal (53,69%) while, high-glucose stimulation decreased the number of bi-hormonal cells (43,3%) (Figure 4D).

### Chronic Treatment With High-Glucose Concentrations Decreases *Proglucagon* mRNA Levels and Proglucagon and Glucagon Protein Expression

**FIGURE 5:**
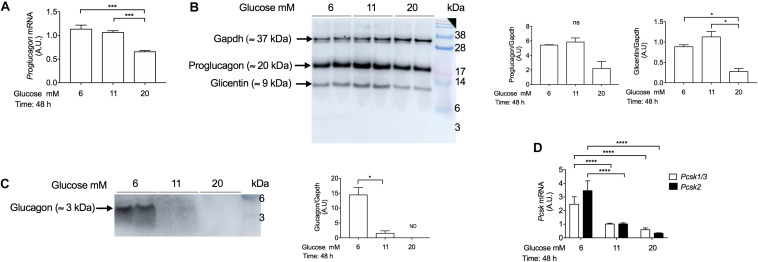
Chronic incubation with high-glucose concentrations reduces *proglucagon* gene and glucagon protein expression in INS-1 cells. **(A)** Real-time PCR analysis of *proglucagon* gene expression in INS-1 cells cultured at 6 mM, 11 mM, and 20 mM glucose for 48 h. Cells incubated with 11 mM glucose were used as controls and were set to an arbitrary value of 1. Gene expression was normalized to *Rlp27* gene. **(B)** Western blot analysis of proglucagon (≈ 20 kDa), glicentin (≈ 9 kDa), and **(C)** glucagon proteins (≈ 3 kDa) expression in INS-1 cells incubated with 6 mM, 11 mM, and 20 mM glucose for 48 h, using the anti-glucagon antibody (Abcam, Cat No. ab92517). Fifteen μg of protein lysates were loaded. Gapdh was used as a loading control. The blot was cut to allow sufficient exposure to detect glucagon. Exposure time for proglucagon proteins: 5 s. Exposure time for Gapdh: 15 s. Exposure time for glucagon: 60 s, capture mode: sensitive (Amersham Imager 600). **(D)** Real-time PCR analysis of *Pcsk1/3 and Pcsk2* gene expression in INS-1 cells cultured with 6 mM, 11 mM, and 20 mM glucose for 48 h. Cells incubated with 11 mM glucose were used as control and were set to an arbitrary value of 1. Gene expression was normalized to *Rlp27* RNA. Data are expressed as means ± SEM. Specific *p*-values: *p < 0.05, ***p < 0.001. One-way ANOVA and Tukey’s *post hoc* test were used. The experiments were performed in duplicate and it was repeated twice.

To determine whether chronic exposure of INS-1 cells to different glucose concentrations may affect *proglucagon* gene expression, we performed qPCR analysis in INS-1 cells cultured with 6 mM, 11 mM, and 20 mM glucose concentrations for 48 h. *Proglucagon* gene expression was decreased in response to chronic treatment with high-glucose (20 mM) compared to 11 mM glucose (Figure 5A). We next investigated the effects of chronically elevated glucose concentrations on proglucagon derived-peptides protein expression by Western blot analysis using a (20%) SDS-Page gel, as described in Supplementary Table 1. Using the anti-glucagon antibody by Abcam (Cat No. ab92517) we observed two bands: a ≈ 20 kDa band, which corresponds to proglucagon, and a ≈ 9 kDa band, which corresponds to glicentin, which were both decreased in high-glucose concentrations (Figure 5B). The decrease in proglucagon protein levels paralleled the decrease in *proglucagon* mRNA levels as measured by q-PCR. Glucagon protein levels were increased in INS-1 cells cultured chronically with low-glucose concentrations (6 mM), but negligible in INS-1 cells cultured chronically with 11 mM and 20 mM glucose concentrations (Figure 5C). This may be explained, at least in part, by the reduced expression of both *Pcsk1* and *Pcsk2* genes (which encode PC1/3 and PC2 proteins) in chronic hyperglycemia (Figure 5D).

### High-Glucose Acutely Stimulates Glicentin and Glucagon Secretion From INS-1 Cells

**FIGURE 6:**
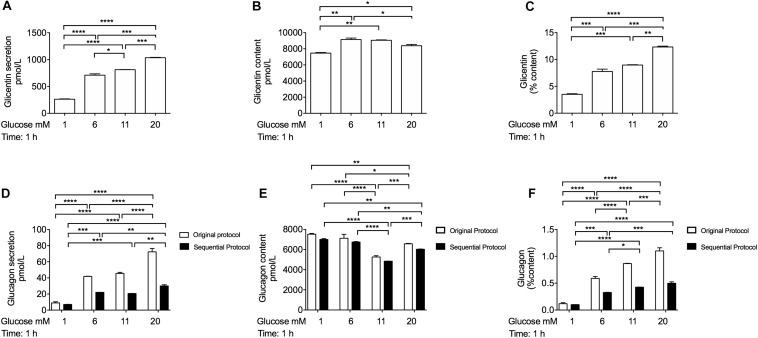
INS-1 cells secret glicentin and glucagon hormones after acute stimulation with high-glucose concentrations. **(A)** Glicentin secretion, **(B)** glicentin content, and **(C)** glicentin secretion expressed as percentage of content from INS-1 cells after acute glucose stimulation measured with the glicentin Elisa kit (Mercodia, Cat No. 10-1275-01). **(D)** Glucagon secretion, **(E)** glucagon content and **(F)** glucagon secretion expressed as percentage of content from INS-1 cells after acute glucose stimulation measured using the glucagon Elisa kit (Mercodia, Cat No. 10-1281-01) with the original protocol (white bars) and with the sequential protocol (black bars). Cells were pre-incubated in 0,5 mM glucose for 1 h and stimulated for an additional hour with different glucose concentrations in KRB buffer. Data are expressed as means ± SEM. Specific values *p < 0.1, **p < 0.01, ***p < 0.001, ****p < 0.0001. Two-way Anova **(A–C)**, one-way ANOVA (D-F), and Tukey’s *post hoc* tests were used. The experiments were performed in triplicate and repeated twice.

Previous studies have shown that PC1/3 and PC2 proteins were co-expressed in bi-hormonal INS-1 cells, suggesting that insulin and proglucagon-derived peptides might share the same secretory pathway (Wang et al., 2015). To confirm this, we measured glicentin and glucagon secretion from INS-1 cells in the same conditions as glucose-stimulated insulin secretion. INS-1 cells were preincubated in 0,5 mM glucose as previously described (Ullrich et al., 1996), for 1 h and sequentially stimulated with different glucose concentrations (1 mM, 6 mM, 11 mM and 20 mM). After stimulation, we observed that glicentin secretion was increased in a glucose-dependent manner (Figures 6A–C), similar to that of insulin (Figure 3E). Under the same experimental conditions, we also observed a similar pattern for glucagon secretion in response to glucose stimulation (Figures 6D–F). Of note, Mercodia reported that glicentin has a 4% cross-reactivity with the glucagon ELISA kit.^2^ As glicentin secretion (Figure 6A) and protein content (Figure 6B) were extremely high in INS-1 cells, we performed Mercodia’s sequential protocol to reduce glicentin cross-reactivity. Although we observed a ≈ 50% reduction in the detection of glucagon secretion in INS-1 cells compared to the original protocol, the secretory pattern for glucagon secretion remained unchanged (Figures 6D–F).

## Discussion

INS-1 cells have been extensively characterized as a mammalian rat cell line, which stores and secretes insulin in response over a wide range of glucose concentrations and are, therefore, widely used to study the mechanisms of insulin secretion (Asfari et al., 1992; Ullrich et al., 1996). In agreement with a previous study, we confirmed that INS-1 cells are heterogeneous, comprising of mature insulin-positive-cells, and immature bi-hormonal cells, which express both insulin and glucagon proteins. Indeed, Wang and colleagues reported that these cells secreted insulin and proglucagon-derived peptides, such as GLP-1, GLP-2, and glucagon, in response to acute glucose-stimulation from the same secreting vesicles. However, they also showed that INS-1 cells do not express transcription factors specific for alpha cell identity such as Pou3f4 or Arx (Bramswig and Kaestner, 2011; Dorrell et al., 2011), thus suggesting that bi-hormonal INS-1 cells are not mature beta-cells (Wang et al., 2015). Indeed, it is known that chronic glucose-stimulation reduces *insulin* gene and protein expression in INS-1 cells (Marie et al., 1993; Olson et al., 1998), however, whether it affects PDPs expression and regulation is unknown. Using cytometry analysis, we observed that the percentage of insulin-positive-cells remained unchanged during chronic exposure to various glucose concentrations. However, high-glucose concentration reduced the percentage of bi-hormonal INS-1 cells and decreased *proglucagon* mRNA levels. A previous study used immunofluorescence-based techniques or ELISA assays to study glucagon expression and secretion in INS-1 cells (Wang et al., 2015). However, immunofluorescence-based techniques show only fluorescently labeled positive-cells, but cannot discriminate between the different proglucagon-derived peptides such as proglucagon, glicentin, and glucagon. Moreover, ELISA assays often have a significant cross-reactivity for proteins that share the same amino acid sequence. Therefore, to discriminate the different proglucagon-derived peptides, we validated the anti-glucagon antibody by optimizing the Western blot procedure to separate and accurately detect low molecular weight proteins. Under these experimental conditions, we observed that INS-1 cells co-expressed proglucagon, glicentin, and glucagon peptides. Although the amount of proglucagon and glicentin was highly expressed, compared to that of glucagon, all three peptides were significantly reduced when INS-1 cells were chronically exposed to high-glucose concentrations. These results are in contrast, to a previous report, showing that chronic hyperglycemia induces glucagon protein expression in fully differentiated mouse beta-cells (Brereton et al., 2014), thus, proposing that the immature status of the bi-hormonal INS-1 cells prohibit the ability to study proglucagon-derived peptides in response to glucose stimulation. Under the same experimental conditions, we observed that *Pcsk1* and *Pcsk2* mRNA levels were decreased, both of which are required for the cleavage of glicentin and glucagon proteins (Marzban et al., 2004; Wang et al., 2015). Notably, the amount of intracellular glicentin content was much higher than that of glucagon, observed by Western blot and ELISA techniques, which may suggest that INS-1 cells express more PC1/3 than PC/2 proteins. Although glicentin is expressed in human pancreatic islets analyzed by Peptide Hormone Acquisition through Smart Sampling Technique-Mass Spectrometry (PHASST-MS) (Taylor S. W. et al., 2013), we also report the expression of glicentin in rat and human primary islets. Indeed, recent studies have shown that some beta-cells co-express insulin, glucagon, and NKX6.1 proteins in pancreatic sections, isolated from type 2 diabetic individuals by immunofluorescence techniques (Spijker et al., 2015; Gutierrez et al., 2017), presuming that glicentin may also be expressed in bi-hormonal beta-cells, under hyperglycemic conditions. However, to date, there is no evidence to suggest a functional role for glicentin in islets since its receptor has not yet been identified (Sinclair and Drucker, 2005).

## Conclusion

We conclude that INS-1 cells are a useful model to study glucose-stimulated insulin secretion, but not glucagon or glicentin peptides. We show that chronic treatment with high-glucose concentrations reduces the bi-hormonal phenotype of these cells. An interesting component of this study is that we optimized the Western Blotting conditions to study proglucagon-derived peptides expression (proglucagon, glicentin, and glucagon) in cell lines and primary islets. It is hoped that this will provide the opportunity for researchers to study glucagon expression and regulation in response to various metabolic stimuli.

## Data Availability Statement

The raw data supporting the conclusions of this article will be made available by the authors, without undue reservation.

## Ethics Statement

The studies involving human participants were reviewed and approved by the Ethical Committee of the University Hospital of Lille, France. The patients/participants provided their written informed consent to participate in this study. The animal study was reviewed and approved by the Ethical Committee of the University of Lille, France.

## Author Contributions

AA-M performed most experiments, analyzed the data, and wrote the manuscript. CS supervised the technical aspects of the project and revised the manuscript. JK-C supervised the islet isolation techniques. JP and FP analyzed and interpreted data, and edited the manuscript. CB supervised the project, analyzed and interpreted data, wrote and edited the manuscript. All authors contributed to the article and approved the submitted version.

## Conflict of Interest

The authors declare that the research was conducted in the absence of any commercial or financial relationships that could be construed as a potential conflict of interest.

## Footnotes

1 https://imagej.nih.gov/ij

2 https://cms.mercodia.com/wp-content/uploads/2019/06/10-1281-01-glucagon-10-ul-dfu-v-3_0.pdf
