# Supplementary figures and images for: Proglucagon-Derived Peptides Expression and Secretion in Rat Insulinoma INS-1 Cells
Source: Front Cell Dev Biol. 2020 Nov 10;8:590763. doi: 10.3389/fcell.2020.590763 (PMC7683504; doi:10.3389/fcell.2020.590763)

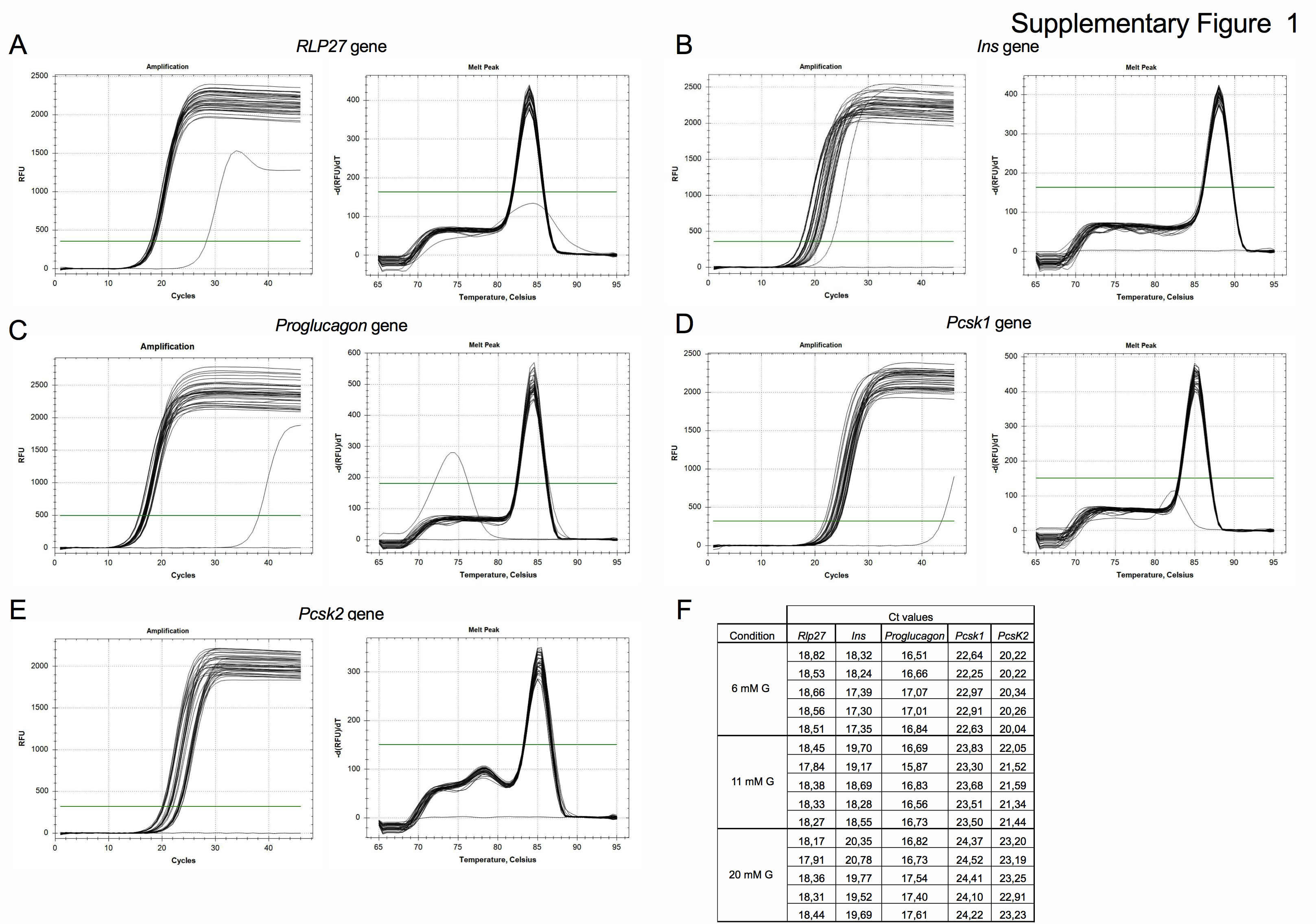

Supplement: Supplementary file 1 [file Image_1.tiff]

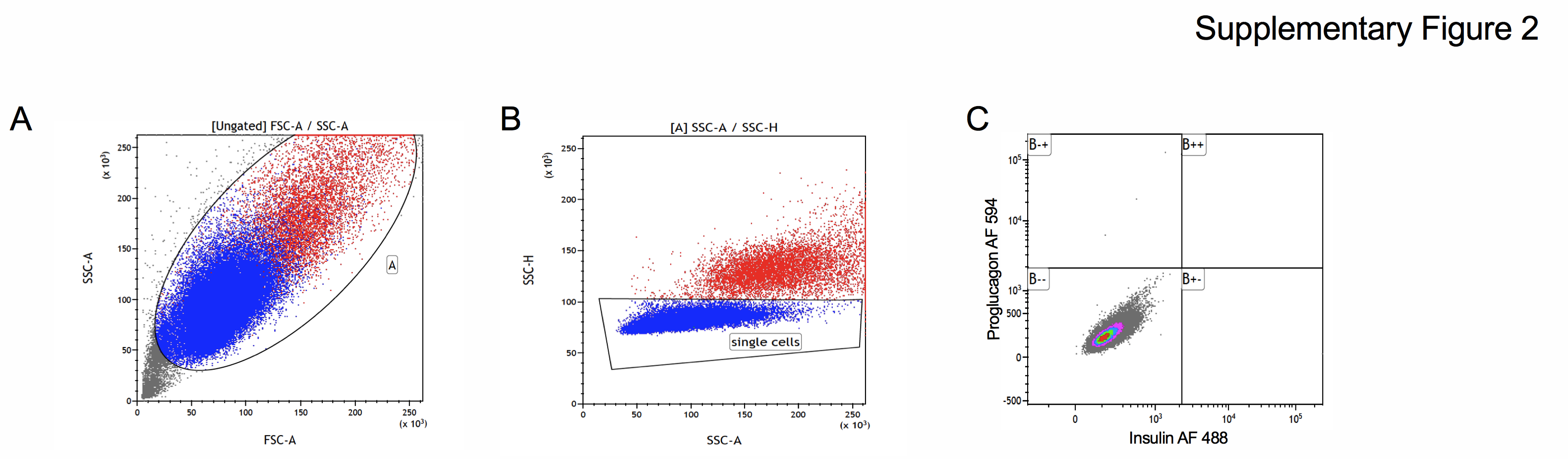

Supplement: Supplementary file 2 [file Image_2.tiff]
