# Supplementary material for: Proglucagon-Derived Peptides Expression and Secretion in Rat Insulinoma INS-1 Cells
Source: Front Cell Dev Biol. 2020 Nov 10;8:590763. doi: 10.3389/fcell.2020.590763 (PMC7683504; doi:10.3389/fcell.2020.590763)
Supplement: Supplementary file 3 [file Table_1.DOCX]

**Supplementary table 1:** Recipe for stacking (4%) and resolving SDS-Page (20%) gels.

|  | Stacking gel  Final volume: 5mL | Resolving Gel  Final volume: 10mL |  |
| --- | --- | --- | --- |
| 40% Acrylamide/Bis solution 37.5:1 | 0,66 mL | 4,4 mL | Biorad, Cat No. 161-0148, China |
| 0,5 M Tris-HCl pH 6.8 | 1,26 mL | - | Trizma Base, Cat No. T6066-5KG, St. Louis USA |
| 1,5 M Tris-HCl pH 8.8 | - | 2,5 mL |  |
| 10% SDS | 50 μL | 100 μL | Sodium dodecyl sulfate, Sigma-Aldrich, Cat No. L4509-500G, St. Louis, USA |
| dH_2_O | 3 mL | 2,9 mL |  |
| TEMED | 5 μL | 5 μL | UltraPure Temed, Invitrogen, Cat No, 15524-010, Carlsbad CA, USA |
| 10% APS | 25 μL | 50 μL | Ammonium persulfate, Sigma-Aldrich, Cat No. A3678-100G, Japan |
