# Supplementary material for: Proglucagon-Derived Peptides Expression and Secretion in Rat Insulinoma INS-1 Cells
Source: Front Cell Dev Biol. 2020 Nov 10;8:590763. doi: 10.3389/fcell.2020.590763 (PMC7683504; doi:10.3389/fcell.2020.590763)
Supplement: Supplementary file 4 [file Table_2.DOCX]

**Supplementary Table 2:** Primer sequences.

| Gene | Species | Sequence primer forward  (5’-3’) | Sequence primer reverse  (5’-3’) |
| --- | --- | --- | --- |
| *Proinsulin* | Rat | cgaagtggaggacccacaag | agtggtgggcctagttgcag |
| *Proglucagon* | Rat | ccattcacagggcacattca | tttcaccagccaagcaatga |
| *Pcsk1/3* | Rat | aggcacctcagcttctgcac | tccaccagagctttggcatt |
| *Pcsk2* | Rat | ccaagttgcagcagaacacg | tgctgcagggccatctttat |
| *Rlp27* | Rat | tacaaccacctcatgcccac | aaacttgaccttggcctccc |
